# Functionalization of 3D Chitinous Skeletal Scaffolds of Sponge Origin Using Silver Nanoparticles and Their Antibacterial Properties

**DOI:** 10.3390/md18060304

**Published:** 2020-06-10

**Authors:** Tomasz Machałowski, Maria Czajka, Iaroslav Petrenko, Heike Meissner, Christian Schimpf, David Rafaja, Jerzy Ziętek, Beata Dzięgiel, Łukasz Adaszek, Alona Voronkina, Valentin Kovalchuk, Jakub Jaroszewicz, Andriy Fursov, Mehdi Rahimi-Nasrabadi, Dawid Stawski, Nicole Bechmann, Teofil Jesionowski, Hermann Ehrlich

**Affiliations:** 1 Institute of Chemical Technology and Engineering, Faculty of Chemical Technology, Poznan University of Technology, Berdychowo 4, 60965 Poznan, Poland; tomasz.g.machalowski@doctorate.put.poznan.pl; 2 Institute of Electronics and Sensor Materials, TU Bergakademie Freiberg, Gustav-Zeuner str. 3, 09599 Freiberg, Germany; iaroslavpetrenko@gmail.com (I.P.); andriyfur@gmail.com (A.F.); 3 Institute of Material Science of Textiles and Polymer Composites, Lodz University of Technology, Zeromskiego 16, 90924 Lodz, Poland; maria.czajka@dokt.p.lodz.pl (M.C.); dawid.stawski@p.lodz.pl (D.S.); 4 Department of Prosthetic Dentistry, Faculty of Medicine and University Hospital Carl Gustav Carus of Technische Universität Dresden, Fetscherstraße 74, 01307 Dresden, Germany; heike.meissner@uniklinikum-dresden.de; 5 Institute of Materials Science, TU Bergakademie Freiberg, Gustav-Zeuner str. 5, 09599 Freiberg, Germany; schimpf@iww.tu-freiberg.de (C.S.); rafaja@ww.tu-freiberg.de (D.R.); 6 Department of Epizootiology and Clinic of Infectious Diseases, Faculty of Veterinary Medicine, University of Life Sciences, Akademicka 13, 20612 Lublin, Poland; achantina@op.pl (J.Z.); beatadziegiel@o2.pl (B.D.); lukaszek0@wp.pl (Ł.A.); 7 Department of Pharmacy, National Pirogov Memorial Medical University, Pirogov str. 56, 21018 Vinnitsa, Ukraine; algol2808@gmail.com; 8 Department of Microbiology, National Pirogov Memorial Medical University, Pirogov str. 56, 21018 Vinnitsa, Ukraine; valentinkovalchuk2015@gmail.com; 9 Materials Design Division, Faculty of Materials Science and Engineering, Warsaw University of Technology, Woloska 141, 02507 Warsaw, Poland; jakubjaroszewicz@wp.pl; 10 Chemical Injuries Research Center, Systems Biology and Poisonings Institute, Baqiyatallah University of Medical Sciences, Tehran 1951683759, Iran; rahiminasrabadi@gmail.com; 11 Faculty of Pharmacy, Baqiyatallah University of Medical Sciences, Tehran 1951683759, Iran; 12 Institute of Clinical Chemistry and Laboratory Medicine, University Hospital Carl Gustav Carus, Technische Universität Dresden, Fetscherstrasse 74, 01307 Dresden, Germany; nicole.bechmann@uniklinikum-dresden.de; 13 Department of Medicine III, University Hospital Carl Gustav Carus, Technische Universität Dresden, Fetscherstrasse 74, 01307 Dresden, Germany; 14 Department of Experimental Diabetology, German Institute of Human Nutrition Potsdam-Rehbruecke, Arthur-Scheunert-Allee 114, 14558 Nuthetal, Germany; 15 German Center for Diabetes Research (DZD), Ingolstaedter Landstrasse 1, 85764 München-Neuherberg, Germany; 16 Center for Advanced Technology, Adam Mickiewicz University, Uniwersytetu Poznańskiego 10, 61614 Poznan, Poland

**Keywords:** chitin, sponges, 3D scaffolds, AgNPs, antibacterial properties, *Aplysina aerophoba*

## Abstract

Chitin, as one of nature’s most abundant structural polysaccharides, possesses worldwide, high industrial potential and a functionality that is topically pertinent. Nowadays, the metallization of naturally predesigned, 3D chitinous scaffolds originating from marine sponges is drawing focused attention. These invertebrates represent a unique, renewable source of specialized chitin due to their ability to grow under marine farming conditions. In this study, the development of composite material in the form of 3D chitin-based skeletal scaffolds covered with silver nanoparticles (AgNPs) and Ag-bromide is described for the first time. Additionally, the antibacterial properties of the obtained materials and their possible applications as a water filtration system are also investigated.

## 1. Introduction

More than 1.2 billion people have no access to clean drinking water [1]. Additionally, 1.8 billion people drinking water from sources susceptible to fecal contamination results in the death of approx. one million children every year [2]. Contaminated potable water, with pathogenic microorganisms (i.e., *Escherichia coli* infection) represents one of the world’s most serious health threats [3]. Consequently, the development of new materials, which are capable of mitigating the risk of bacterial contamination in water needs to be an ongoing, crucial area of research [4].

Filtration is a widely used method of treating water and recent, numerous attempts aim to develop effective antibacterial composite-based filtration materials [5,6]. Promising examples with respect to their potential application include synthetic polymers (e.g., polypropylene, polyurethane, polyacrylonitrile) [7,8,9], natural materials (e.g., chitin, chitosan, cellulose, collagen) [10,11,12] as well as carbon-based composites [13,14], which have been covered with silver nanoparticles (AgNPs) using diverse techniques. The use of silver compounds to disinfect water and the procedure’s resulting death of fungi, molds, bacteria and various spores has been documented since ancient times, as cited by Atiyeh [15]. Today, it is a proven fact that direct contact with silver inactivates cells and microorganisms [16]. The mechanism of action of silver involves the inhibition of microbial respiration through binding of metal particles to the bacterial cell membranes [17]. Consequently, silver impairs the microbial respiratory system. The fundamental factors affecting the superior antimicrobial properties of silver-based composites are the size of Ag particles and their solid phase surface development. As recently reported, the “antimicrobial activity of the smaller Ag nanoparticles may be several orders of magnitude greater than that of the corresponding bulk solid” [18]. Thus, it is not surprising that AgNPs are most commonly used in many antibacterial products to protect health and improve the quality of life [19]. 

**Figure 1:**
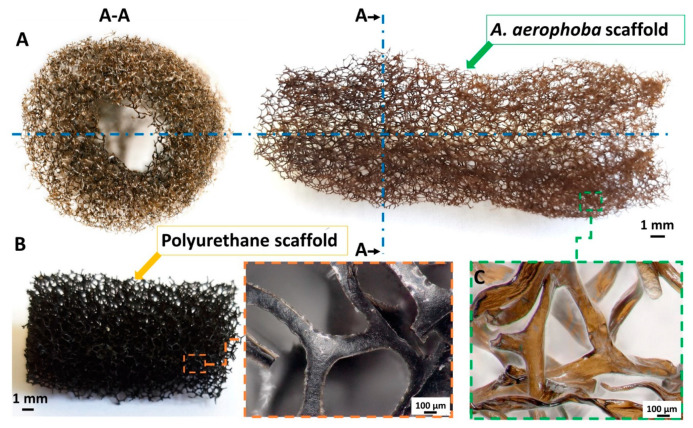
The optical representation of a decellularized *Aplysina aerophoba* demosponge 3D chitinous skeletal scaffold (**A**). Representation of the cross-section (**A**-**A**). Polyurethane (PU) scaffolds, traditionally used as water filter material, with high magnification of the fibers (**B**). Microscopic representation of the isolated chitinous skeletal scaffold (**C**) shows high structural similarity to the commonly used PU-based filtration material (**B**). The light brownish color is due to the presence of brominated compounds naturally occurring in the skeletal fibers of the sponge.

Metallization of chitin [20,21], as one of the most abundant structural polysaccharides in nature [22,23], with production in oceans measuring approx. 10^12–14^ tons per year [24], remains a solid trend. Chitin is synthesized by a broad assortment of organisms representing different taxonomic groups, mostly crustaceans [25,26,27,28,29] and insects [30,31,32,33,34,35]. Nowadays, the functionalization of naturally predesigned chitinous scaffolds with a 3D architecture attracts particular attention [36,37,38,39,40,41]. In this article, the unique skeletal chitin-based 3D scaffolds (Figure 1) isolated from the cultivated under marine farming *Aplysina aerophoba* marine demosponge were used for the first time as a basic construct for fabrication of an antibacterial water filter. This biomaterial was modified by silver nanoparticle deposition using chemical reduction of silver nitrate and the antibacterial action was investigated. 

## 2. Results 

**Figure 2:**
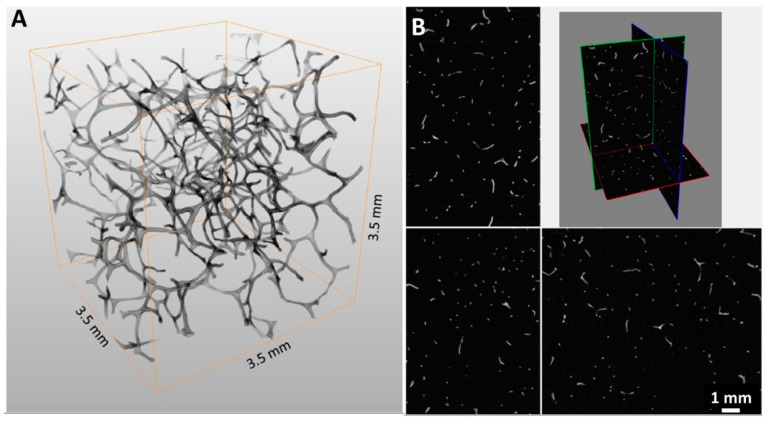
The 3D model (**A**) and cross-sections (**B**) of the 3D chitin–Ag/AgBr composite scaffold obtained by micro-CT.

Chitinous 3D scaffolds represent an intriguing alternative to synthetic analogues [20,21,36]. Due to the high porosity (Figure 2) and structural similarity of the poriferan 3D chitinous scaffolds to synthetically produced porous foams, this biological material is particularly interesting for filtration applications. Based on micro-focused X-ray tomographic (micro-CT) analysis (Figure 2), the porosity of a chitinous scaffold isolated from *A. aerophoba* demosponge was estimated at 98.5% (see Table S1). 

**Figure 3:**
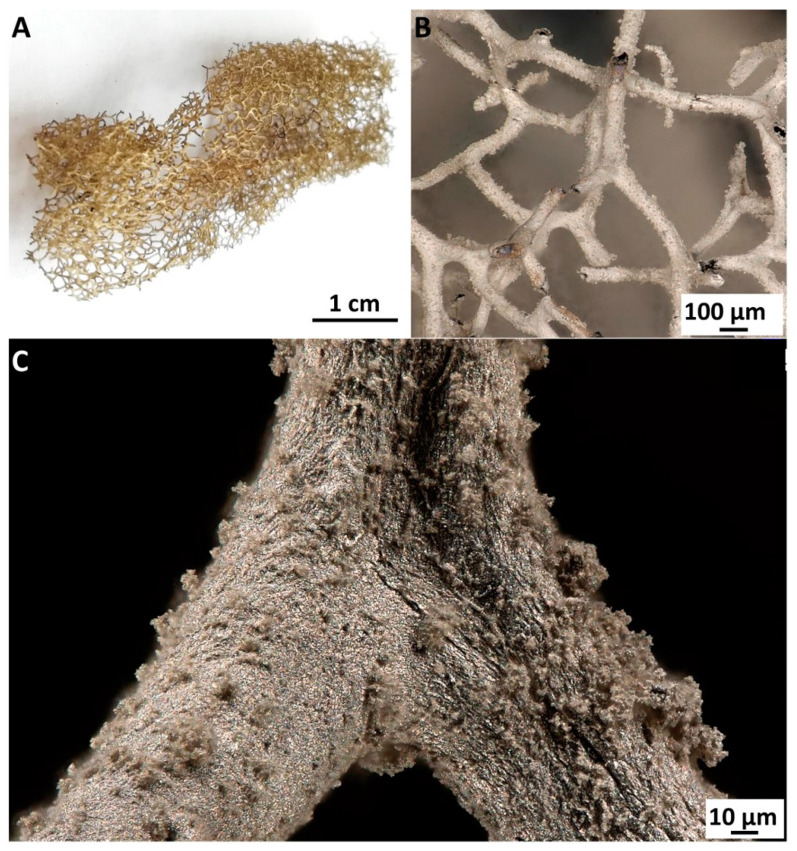
The 3D chitinous scaffolds isolated from the *A. aerophoba* sponge resemble their microarchitecture being covered with nanoparticles of Ag/AgBr (**A**,**B**). The stereomicroscopy image represents the existence of the tightly bound metallized layer, also taken after 30 min of sonication (**C**).

The alkaline environment of the chemical reduction of AgNO_3_ promoted the additional release of bromine-derived compounds originally located within the fiber of the chitinous scaffold isolated from *A. aerophoba* demosponge [37]. The clearly visible metallic layer obtained after metallization with Ag is strongly bound to the chitinous fibers even after ultrasonic treatment (Figure 3).

**Figure 4:**
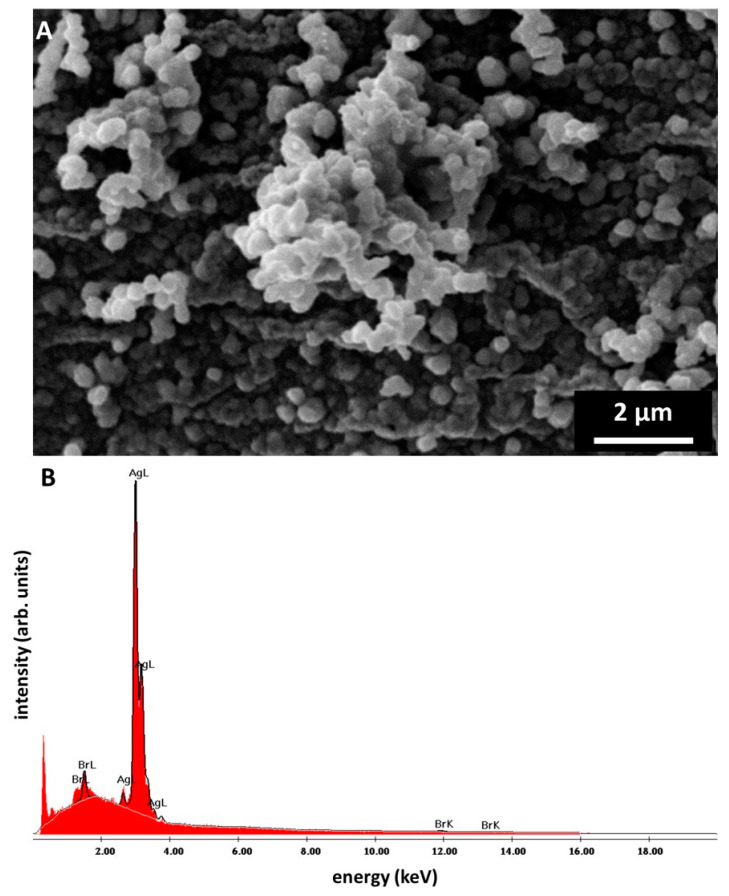
(**A**) SEM image of the surface of the skeletal chitinous scaffold isolated from *A. aerophoba* demosponge covered by the layer of silver/silver bromide nanoparticles. EDX analysis confirms the presence of both Ag and Br within these nanoparticles (**B**). This is in good agreement with the XRD data obtained for the same sample (see Figure 5).

**Figure 5:**
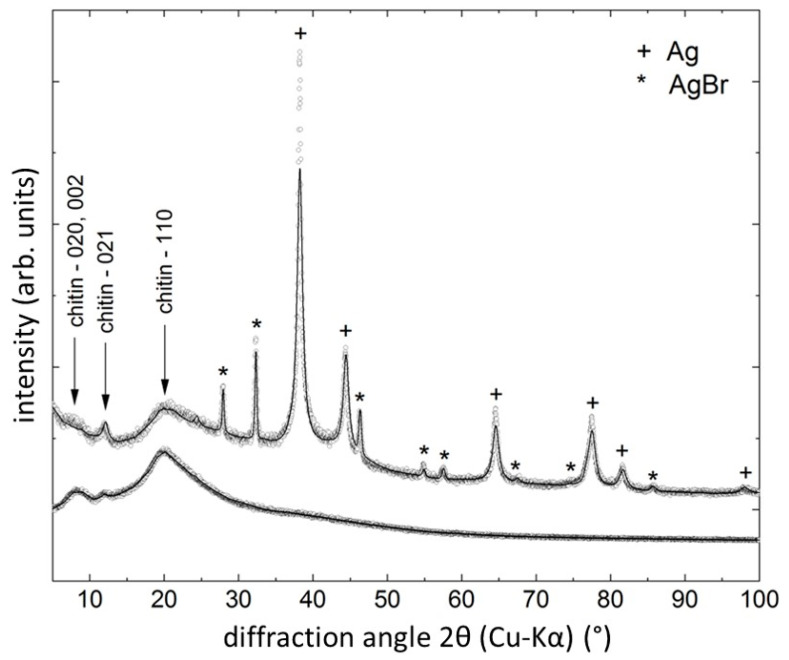
X-ray diffraction patterns (small circles: measured intensities; lines: refinement) of the pure sponge chitin (lower signal intensity) and a chitin sample tightly covered with the Ag and AgBr nanoparticles (upper signal intensity). The sharp diffraction lines in the diffraction pattern belong either to Ag or to AgBr, as indicated.

Ag/AgBr nanoparticles tend to create the spherical-shaped aggregates represented in Figure 4A. The SEM image in Figure 4A shows the chitinous fiber completely covered by Ag/AgBr aggregates, which are exclusively deposited on the surface of the fibers. The highest fraction of aggregates composed of nanoparticles, which constitute approx. 42%, contains particles with a diameter range of 300–400 nm (see Figure S2). At the surface of chitinous fiber, the creation of nanostructured agglomerates with dimensions up to two μm is also observable. EDX-based analysis of the surface of the metallized *A. aerophoba* chitinous scaffold confirms domination of Ag and Br (see Figure 4B and Table S2). Obtained data explains the identification of the Ag-bromide phase within the metallized layers using XRD analysis (see Figure 5).

The X-ray diffraction patterns of the skeletal chitin sample before and after reaction with AgNO_3_ solution are shown in Figure 5. In both cases, the crystal structure of chitin (see [42]) is lost due to pretreatment of the samples before XRD. However, some remainders of the chitin structure are still visible, i.e., the diffraction maximum 021 at 2θ ≈ 12° and the ‘hump’ beginning with the 110 reflection near 2θ ≈ 20°. The XRD line from chitin are marked in Figure 5. The chitin sample treated with AgNO_3_ also shows, in addition to the remaining features of the chitin diffraction pattern, peaks of Ag (PDF# 04-004-6434) and AgBr (PDF# 00-006-0438). The presence of metallic Ag confirms the applicability of the synthesis route for the creation of Ag nanoparticles on chitin surface. The Rietveld analysis revealed the Ag lattice parameter of *a* = (4.088 ± 0.001) Å, which is practically identical to the tabulated value of *a* = 4.089 Å, and a crystallite size of *D*_iso_ = (13 ± 2) nm, confirming the nanocrystalline character of the particles. After Br was identified by the EDX analysis, the remaining peaks in the XRD pattern were successfully assigned to AgBr crystallizing in space group Fm$\bar{3}$m, with a lattice parameter of a = (5.555 ± 0.002) Å. Due to the treatment of the scaffolds with AgNO_3_, we have also checked for the presence of silver oxides, nitrides and chloride but found no positive match among the database entries. The amount of metallic Ag is approx. 75 vol.%; the amount of AgBr is approx. 25 vol.%, as indicated by the quantitative XRD phase analysis.

The assessment of the antibacterial activity of the prepared composite was based on the agar diffusion method. As shown in Figure S3, the inhibition zone of the 3D chitin–Ag/AgBr composite in respect to *E. coli* is greater than that of chitinous skeletal scaffolds before metallization (see Table 1), which indicates the superior antibacterial properties of the created 3D construct. The obtained material contributed to wide zones of inhibition, and the mean value was estimated at 23 and 24 mm for *Escherichia coli and Bacillus subtilis,* respectively (see Figure S3). Interesting results were also obtained for the chitinous scaffold before silver coating with respect to the *E. coli*. The chitinous scaffold used as control was isolated by the method described in Section 4.1. It is mainly composed of Br-containing chitin [37], which is originally responsible for the resistance of this verongiid sponge against predatory microorganisms from marine environments. In this case, the zone of inhibition was 18 mm. However, this effect was not observed in the case of Gram-positive bacterium *B. subtilis* that was comparatively used in this study. Interestingly, the commercially available antibacterial material Suprasorb^®^ A + Ag, which was also used for comparative purposes, did not contribute to any zones of inhibition against both bacterial strains studied. 

**Figure 6:**
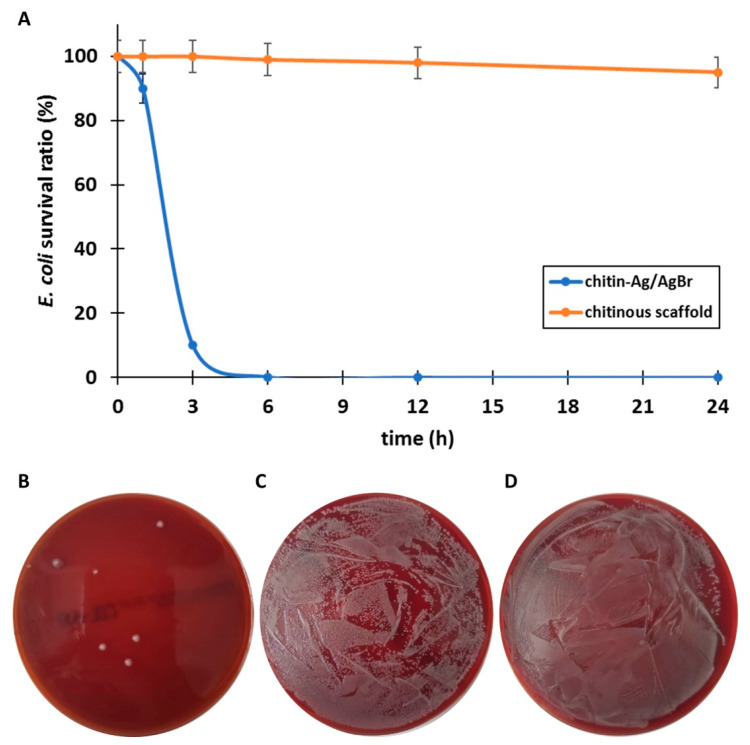
The dynamics of the degradation of live bacteria colonies shown over time, by test tube assay, using a 3D chitin–Ag/AgBr scaffold and a chitinous scaffold before Ag coating, with 5% error bars (**A**). Only seven *E. coli* bacteria colonies survived after 24 h of testing using a chitin–Ag/AgBr scaffold (**B**). Both Br-containing chitinous scaffolds before silver coating (**C**) and commercially available material Suprasorb^®^ A + Ag (**D**) did not show antibacterial activity against *E. coli* even after 24 h.

The test tube assay additionally confirmed the antibacterial properties of the chitin–Ag/AgBr scaffold (Figure 6). It was observed that the percentage of the bacteria survival decreased with 24 h and more than 99.9% of the initial bacterial CFU was eliminated (for details, see Table 1 and Figure 6). The rapid increase in *E. coli* degradation was observed between 1 and 3 h (Figure 6A). In contrast to the agar diffusion method, the Br-containing chitinous scaffold before silver coating did not show visible antibacterial potential against *E. coli* even after 24 h.

Results obtained after filtration (Figure S4) clearly indicate that 3D chitin–Ag/AgBr scaffolds possess antibacterial properties against the *E. coli* (ATCC^®^ 25922) strain. In experiments, a superior inhibitory effect was observed after 6 h of filtration with this material. After 24 h, only one colony of this bacterium survived (Figure S4B). Filtration with the Br-containing chitinous scaffold before Ag coating did not cause an observable changes in the number of bacteria. In future, the influence of a diverse chitin–Ag/AgBr filter density has to be examined in relation to enhance antibacterial effect. No increase in absorbance at 419 nm indicated that the silver nanoparticle-based layer was stable on the developed 3D water filter and was not washed away by water flow even after 24 h [43].

## 3. Discussion 

Recently observed rapid development of nanotechnology, especially in the fields of bioinspired materials science and biomimetics, is strictly related to searching for new methods for synthesis of effective hybrid materials and biocomposites with designed properties [44]. Until the discovery of chitin in fibrous skeletons of some verongiid demosponges in 2007 [45], as well as in the verongiid demosponge species *A. aerophoba* in 2010 [46], the commonly accepted opinion was that skeletal fibers of these demosponges are made of a proteinaceous, biological material called spongin [47]. However, this erroneous assumption was experimentally proved based on the solubility of spongin in alkaline solutions, which was not observed with chitin resistant to such chemical conditions [20,36]. It is the resistance of chitin to an alkaline medium up to a concentration of 10% that allows us to look for key ways to use it in specific chemical reactions, including silver metallization, as described in this work. For example, the implementation of such a reaction using spongin-based matrices would not have been possible.

Chitin of poriferan origin also possess characteristic features such as thermostability up to 400 °C [41,48,49], cytocompatibility [50,51] and microporosity [36,52]. Recently, practical applications of demosponge chitinous scaffolds were reported for tissue engineering [39,50,51,53], uranium adsorption [54], bioelectrometallurgy and extreme biomimetics [21] as well as for the photodegradation of organic dyes [49]. Here, we describe, for the first time, antibacterial properties of composite material in the form of 3D chitin-based skeletal scaffolds covered with AgNPs and Ag-bromide. The formation of Ag-bromide on the surface of this specific chitin is due to the presence of bromine in the chitin skeleton of these sponges. We suggest that this compound is formed as a result of alkaline extraction of bromine-containing compounds in the presence of silver ions.

Originally, diverse brominated derivatives (mostly bromotyrosines) [37] are located in the skeletal fibers being intercalated into chitinous layers. They represent an effective form of biochemical defense against harmful pathogens, which constantly fall inside the verongiid sponge, which filters the surrounding water to extract the appropriate feed (i.e., viruses, bacteria, organic micro debris). These unique defense strategies, based on Br-containing secondary metabolite production, allowed this organism to survive more than 500 millions of years [37]. Today, bromotyrosines are recognized as multi-targeted marine drugs with broad fields of applications (i.e., as antitumorigenic and antimetastatic agents [55,56]). In order to isolate pure chitinous scaffolds from verongiid demosponges, diverse methodological approaches based on chemical, electrochemical and enzymatic treatments were reported (see for overview [36,57]). Nonetheless, the alkali treatment of verongiid skeletons is most commonly used [20,36,40] and can be regulated with respect to residual bromine concentration in the chitinous matrices. In our case, secondary metabolite compounds present within scaffolds become a great source of natural bromine. Previously, superb antibacterial properties of Ag [6,16,58] and AgBr [59,60,61] nanoparticles were already reported, but as separate substances and not within composite materials. 

In order to determine antibacterial properties of the designed 3D chitin–Ag/AgBr composite, the agar diffusion method as well as test tube assay was carried out. Moreover, a simple prototype of the filtration set was proposed to assess the determination ability of 3D chitin–Ag/AgBr scaffolds in terms of *E. coli* inactivation. Based on the obtained data, appropriative antibacterial properties against the *E. coli* strain were reported here. Only one bacteria colony from 10^6^ CFU/µL survived after 24 h of filtration. In the 3D composite scaffold developed in this study, under ambient conditions, the content of Ag is approx. 75 vol.% and the amount of AgBr is approx. 25 vol.%. This distinguishes the method described in this study from the metallization of chitin in harsh chemical conditions. For example, previously, poriferan chitin was effectively used as a template for solvothermal and hydrothermal conditions according to an extreme biomimetic approach [41,44,48,49,62]. Element oxide-based composites such as chitin-SiO_2_ [62,63], chitin-GeO_2_ [41], chitin-ZrO_2_ [48,64], chitin-ZnO [65], and chitin-hematite [66] were synthesized under hydrothermal conditions between 65 and 185 °C. 

## 4. Materials and Methods 

### 4.1. Chitin Scaffold Isolation

Air-dried specimens of cultivated *Aplysina aerophoba* (Nardo, 1833) marine demosponges were purchased from BromMarin GmbH (Freiberg, Germany). Chitinous scaffolds were isolated by chemical treatment of specimens in 2 days as follows. At the first stage, selected fragments of *A. aerophoba* skeleton were immersed in pure distilled water at 80 °C for 24 h to remove water-soluble compounds and cells, which were disrupted due to osmotic shock. In the next step, cell-free skeletons, were immersed in 20% acetic acid at room temperature over 4 h in order to remove calcium carbonates. The prefinal stage included treatment with 2.5 M NaOH solution for 6 h at 37 °C, for deproteinization and partial depigmentation. Finally, Br-containing skeletal 3D chitinous scaffolds were neutralized by distilled water and stored at 4 °C.

### 4.2. Fabrication of Silver-Coated 3D Chitinous Scaffolds

In order to obtain AgNps on the surface of skeletal chitinous scaffolds isolated from the cultivated *A. aerophoba* demosponge, chemical reduction of AgNO_3_ was carried out. For this purpose, a selected fragment of the skeletal scaffold (10 × 30 mm) was immersed in 30 mL of 1 M AgNO_3_ solution for 1 h at room temperature. Then, 15 mL of 0.8 M NaOH was added to the above mixture and formation of a precipitate was observed. In the next step, a concentrated ammonia solution was added dropwise until total precipitate dissolution was achieved. After 15 min, 1 mL of ethanol and 25 mL of a mixture of 0.08 M glucose and 0.04 M citric acid were added. The obtained construct was washed several times by deionized water and treated by ultrasound (60 kHz, 300 W) for 30 min at room temperature in order to remove non-attached nanoparticles of silver. 

### 4.3. Characterization of Obtained Materials 

#### 4.3.1. Digital Microscopy

Corresponding samples were observed and analyzed by an advanced imaging and measurement system consisting of a Keyence VHX-6000 digital optical microscope (Osaka, Japan) and the swing-head zoom lenses VH-Z20R (magnification up to 200×) and VH-Z100UR (magnification up to 1000×). 

#### 4.3.2. Micro-CT Analysis 

Scaffolds were scanned using a micro-focused X-ray tomographic system (MICRO XCT-400, Xradia–Zeiss, Pleasanton, CA, USA) at 40 kV and 200 μA. For each sample, 1500 projection images were recorded with an exposure time of 12 sec and a magnification objective of 20×. The volume was reconstructed with the instrument software and was then exported to the CTAn (Bruker Billerica, MA, USA) program for further 3D image analysis. Voxel size was the same for all samples (2 × 2 × 2 μm).

#### 4.3.3. Infrared Spectroscopy 

Attenuated total reflectance Fourier transform infrared spectroscopy (ATR-FTIR), was used for the qualitative characterization and identification of the isolated materials. The samples were analyzed by a Nicolet 210c spectrometer (Thermo Fisher Scientific, Waltham, MA, USA).

#### 4.3.4. UV–VIS Spectroscopy 

The absorption at 419 nm was recorded with a spectrophotometer (SPECORD S10, Carl Zeiss, Germany). Measurements were recorded for determination of the presence of silver nanoparticles in the solution after filtration [43]. A quartz cuvette was used with a path length of 1 cm (Quartz SUPRASIL^®^, Hellma Analytics, Müllheim, Germany) and operated at a resolution of 5 nm.

#### 4.3.5. Scanning Electron Microscopy (SEM) and Energy-Dispersive X-ray Spectroscopy (EDX)

Samples were prepared for analysis by freeze-drying, followed by covering with Au using a Cressington Sputter Coater S150B (BOC Edwards, Wilmington, MA, USA). Scanning electron microscopy was performed using a Hitachi S-4700-II (Hitachi, Ltd., Tokyo, Japan) field emission microscope. The elements were analyzed by energy-dispersive X-ray spectroscopy in the EDX analysis system from EDAX and a XL30ESEM Philips scanning electron microscope (Philips, Amsterdam, The Netherlands). Observations were carried out under high vacuum, 20 kV voltage, and 6500× and 8000× magnification. The particle diameter was determined from 100 measurements using representative micrographs by the software (ImageJ, National Institutes of Health, Bethesda, MD, USA). 

#### 4.3.6. X-ray Diffraction 

X-ray diffraction measurements were performed with the purpose of phase identification and quantification. A FPM RD7 diffractometer equipped with a sealed X-ray tube with Cu anode operated at 40 kV/30 mA was used. The diffractometer worked in the symmetrical diffraction geometry and the powderized sample was fixed with ethanol on a Si[510]-cut zero-background holder. The diffracted beam passed a graphite monochromator to eliminate unwanted radiation components before being registered by a proportional counter. The measurements covered the angular range from 5° to 150° 2θ, with a step size of 0.02° and a dwell time of 12 s per point. Data analysis was performed by database search (ICSD PDF-4+) with the Panalytical HighScore+ program. Rietveld refinement of the diffraction patterns was performed by the MAUD software package [67].

### 4.4. Antibacterial Activity Studies

#### 4.4.1. Determination of the Zone of Inhibition

The chitin–Ag/AgBr (1) (Figure S2) composite scaffolds were tested for their antibacterial activity using an agar diffusion method. For this purpose, 0.01 g of sample was prepared. For comparison, chitinous scaffold before silver coating (2) and commercially available antibacterial wound dressing Suprasorb^®^ A + Ag (3) were used. *E. coli* (ATCC^®^ 25922) and *B. subtilis* B9 (Collection of Department of Epizootiology and Clinic of Infectious Diseases, University of Life Sciences in Lublin, Poland) were taken as model Gram-negative and Gram-positive bacteria (1.5 on the McFarland scale). Bacteria strain was applied with agar Mueller–Hinton medium. Afterwards, plates were incubated at 37 °C for 24 h. The diameter of inhibitory zone surrounding material pieces was measured in mm for each specimen. Tests performed on three agar plates (for each material) determined a mean value.

#### 4.4.2. Test Tube Antibacterial Assay

For the test tube assay, the *E. coli* ATCC^®^ 25,922 suspension (0.8 on the McFarland scale, approx. 10^6^ CFU/µL) in 0.9% sodium chloride was prepared in sterilized test tubes. An amount of 0.01 g of sample (No. 1–3 see Section 4.4.1.) was put into the separated test tubes and immersed in 1.5 mL of suspension described above at 37 °C for 1, 3, 6, 12 and 24 h. The scaffolds were taken out from the test tubes. Then, 100 μL of suspension was plated on Columbia LabAgar plates + 5% sheep blood and incubated at 37 °C for 24 h for determination of number of survive bacterial colonies from the control sample. These measurements were repeated three times and a mean value was evaluated.

#### 4.4.3. Determination of Antibacterial Properties—Filtration Test

The antibacterial activity of the obtained chitin–Ag/AgBr (1) was also determined using the filtration method. The *E. coli* ATCC^®^ 25,922 suspension (0.8 on the McFarland scale, approx. 10^6^ CFU/µL) was kept in a storage 1000 mL container filled with 0.9% sodium chloride and filtered at a flow rate of 330 mL/min. The suspension was pumped into the sterilized filtration cartridge, which consists of a 0.5 g of filter inside 50 cm^3^ falcon tubes (Figure S1). Changes in the survival rate of the bacteria colony were determined for 1, 3, 6, 12 and 24 h analogous to the test described above.

## 5. Conclusions

The fundamental difference between chitin-based 3D scaffolds originating from verongiid sponges and other, previously reported chitinous matrices is the possibility to regulate the content of Br during the preparation process by changing the time of alkaline treatment of original skeletal 3D constructs. Therefore, the experimental approach described in this study enables unique nanostructured Ag/AgBr composite in the form of a nanolayer, which remains strongly bound to the surface of the organic matrix and is responsible for selective antibacterial activities. The results open the path to using chitin-based skeletal matrices in the form of acellular scaffolds. Due to the natural ability of the *A. aerophoba* demosponges to regenerate their skeletons and to grow at low depths under marine farming conditions at large scales, their potential for applications in bioinspired materials science and technologies increases dramatically. Future research, dedicated to the optimization of such naturally derived, already prefabricated materials using other metal ions as well as alternative reductants (e.g., lignosulfonates) also for special biomedical applications is strongly indicated.

## Figures and Tables

**Table 1:** Mean zone of inhibition for both strain (mm) and number of survived *Escherichia coli* strains after 24 h of test tube assay.

Material	Agar Diffusion Method		Test Tube Test	
	*E. coli* (mm)	*B. subtilis* (mm)	*E. coli* (CFU/100 μL)	*% of Reduction*
Chitin–Ag/AgBr scaffold	23	24	7	99.9
Chitin-based scaffold	18	0	∼10^6^	0
Suprasorb^®^ A + Ag Control	0 0	0 0	∼10^6^ ∼10^6^	0 0

## Supplementary Materials

The following are available online at https://www.mdpi.com/1660-3397/18/6/304/s1, Figure S1: The protype of the filtration set used in this study. Figure S2: Percentage of Ag/AgBr aggregates in individual fractions as a function of diameter ranges. Figure S3: Agar diffusion method results. The antimicrobial activity of chitin–Ag/AgBr scaffolds (1), chitinous scaffold before metallization (2) against *E. coli* (A). Lack of antimicrobial activity represent by Suprasorb^®^ A + Ag (3) against *E. coli* (A). The antimicrobial activity of chitin–Ag/AgBr scaffold (1) against *B. subtilis* (B). Figure S4: Dynamics of the degradation of live bacteria colonies as a function of time of filtration with 5% error bars (A). Filtration clearly indicates that chitin–Ag/AgBr scaffolds possess antibacterial properties against the *E. coli* (ATCC^®^ 25922) strain (blue line). Br-containing chitinous scaffolds before AgNP coating did not reflect any antibacterial effect. The number of survived bacteria colonies was uncountable, even after 24 h (orange line). Only one colony of *E. coli* survived after 24 h of filtration using chitin–Ag/AgBr scaffold (B). Chitinous scaffold before silver coating did not show antibacterial potential against *E. coli* even after 24 h (C). Table S1: Results of 3D quantitative analysis (micro-CT). Table S2. Results of local chemical analyses (SEM-EDX) of selected areas of the *A. aerophoba* chitinous scaffold after metallization.
