# Supplementary material for: Functionalization of 3D Chitinous Skeletal Scaffolds of Sponge Origin Using Silver Nanoparticles and Their Antibacterial Properties
Source: Mar Drugs. 2020 Jun 10;18(6):304. doi: 10.3390/md18060304 (PMC7345230; doi:10.3390/md18060304)
Supplement: Supplementary file 1 [file marinedrugs-18-00304-s001.pdf]

Supplementary Materials

Functionalization of 3D Chitinous Skeletal Scaffolds of Sponge Origin Using Silver Nanoparticles and Their Antibacterial Properties

Tomasz Machałowski [1,2], Maria Czajka [3], Iaroslav Petrenko [2], Heike Meissner [4], Christian Schimpf [5], David Rafaja [5], Jerzy Ziętek [6], Beata Dzięgiel [6], Łukasz Adaszek [6], Alona Voronkina [7], Valentin Kovalchuk [8], Jakub Jaroszewicz [9], Anrdiy Fursov [2], Mehdi Rahimi-Nasrabadi [10,11], Dawid Stawski [3], Nicole Bechmann [12,13,14,15], Teofil Jesionowski [1,*] and Hermann Ehrlich [2,16,*]

[1] Institute of Chemical Technology and Engineering, Faculty of Chemical Technology, Poznan University of Technology, Berdychowo 4, Poznan 60965, Poland; tomasz.g.machalowski@doctorate.put.poznan.pl (T.M.)
[2] Institute of Electronics and Sensor Materials, TU Bergakademie Freiberg, Gustav-Zeuner str. 3, Freiberg 09599, Germany; iaroslavpetrenko@gmail.com (I.P.); andriyfur@gmail.com (A.F.)
[3] Institute of Material Science of Textiles and Polymer Composites, Lodz University of Technology, Zeromskiego 16, Lodz 90924, Poland; maria.czajka@dokt.p.lodz.pl (M.C.); dawid.stawski@p.lodz.pl (D.S.)
[4] Department of Prosthetic Dentistry, Faculty of Medicine and University Hospital Carl Gustav Carus of Technische Universität Dresden, Fetscherstraße 74, Dresden 01307, Germany; heike.meissner@uniklinikum-dresden.de (H.M.)
[5] Institute of Materials Science, TU Bergakademie Freiberg, Gustav-Zeuner str. 5, Freiberg 09599, Germany; schimpf@iww.tu-freiberg.de (C.S.); rafaja@ww.tu-freiberg.de (D.R.)
[6] Department of Epizootiology and Clinic of Infectious Diseases, Faculty of Veterinary Medicine, University of Life Sciences, Akademicka 13, Lublin 20612, Poland; achantina@op.pl (J.Z.); beatadziegiel@o2.pl (B.D.); ukaszek0@wp.pl (Ł.A.)
[7] Department of Pharmacy, National Pirogov Memorial Medical University, Pirogov str. 56, Vinnitsa 21018, Ukraine; algol2808@gmail.com (A.V.)
[8] Department of Microbiology, National Pirogov Memorial Medical University, Pirogov str. 56, Vinnitsa 21018, Ukraine; valentinkovalchuk2015@gmail.com (V.K.)
[9] Materials Design Division, Faculty of Materials Science and Engineering, Warsaw University of Technology, Woloska 141, Warsaw 02507, Poland; jakubjaroszewicz@wp.pl (J.J.)
[10] Chemical Injuries Research Center, Systems Biology and Poisonings Institute, Baqiyatallah University of Medical Sciences, Tehran 1951683759, Iran; rahiminasrabadi@gmail.com (M.R.-N.)
[11] Faculty of Pharmacy, Baqiyatallah University of Medical Sciences, Tehran 1951683759, Iran;
[12] Institute of Clinical Chemistry and Laboratory Medicine, University Hospital Carl Gustav Carus, Technische Universität Dresden, Fetscherstrasse 74, Dresden 01307, Germany; nicole.bechmann@uniklinikum-dresden.de (N.B.)
[13] Department of Medicine III, University Hospital Carl Gustav Carus, Technische Universität Dresden, Fetscherstrasse 74, Dresden 01307, Germany;
[14] Department of Experimental Diabetology, German Institute of Human Nutrition Potsdam-Rehbruecke, Arthur-Scheunert-Allee 114, Nuthetal 14558, Germany;
[15] German Center for Diabetes Research (DZD), Ingolstaedter Landstrasse 1, München-Neuherberg 85764, Germany;
[16] Center for Advanced Technology, Adam Mickiewicz University, Uniwersytetu Poznańskiego 10, Poznan 61614, Poland;
* Correspondence: Teofil.Jesionowski@put.poznan.pl (T.J.); Hermann.Ehrlich@esm.tu-freiberg.de (H.E.)

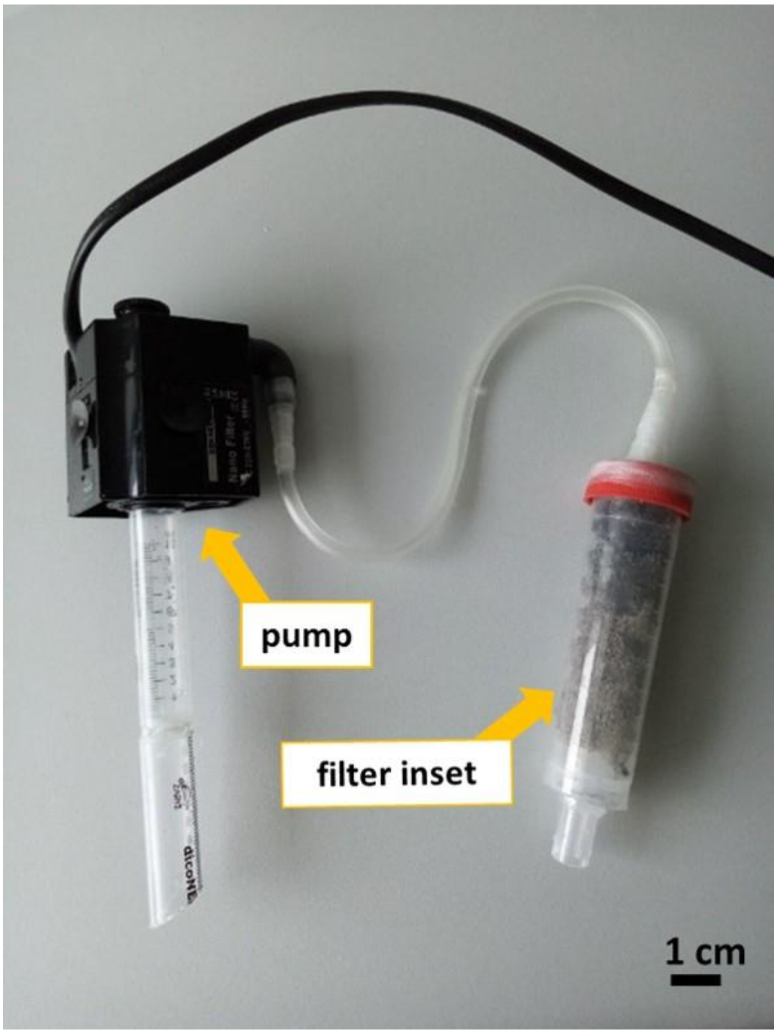

Figure S1. The prototype of the filtration set used in this study.

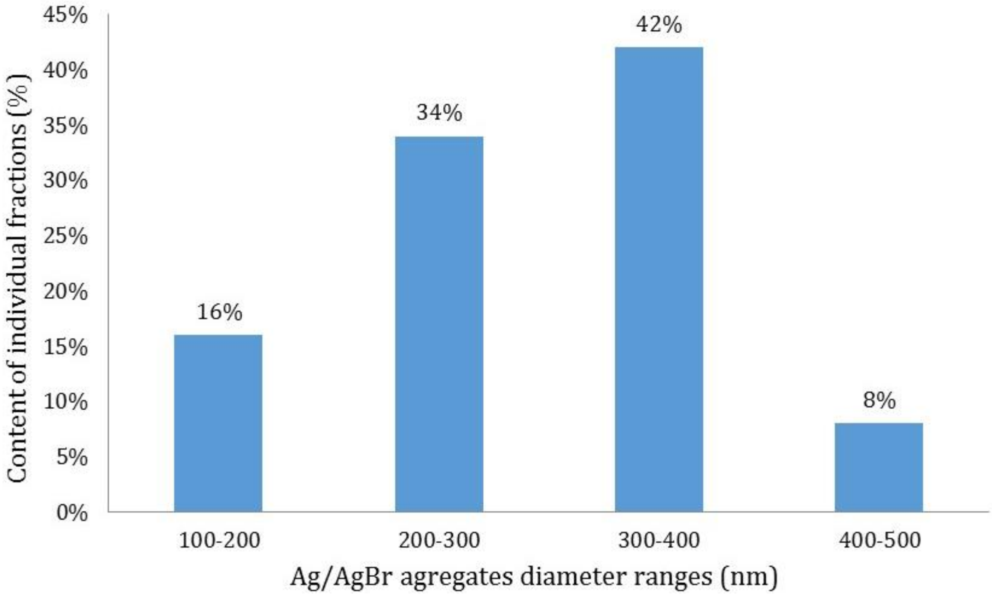

Figure S2. Percentage of Ag/AgBr nano-aggregates in individual fractions as a function of diameter ranges.

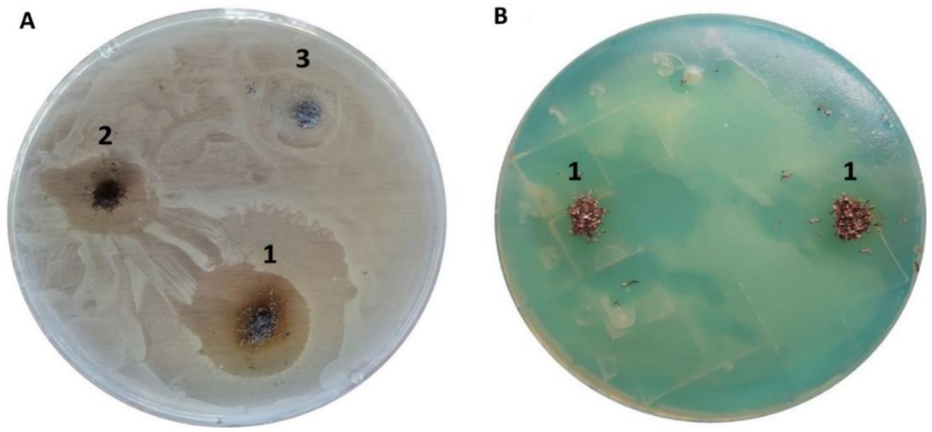

Figure S3. Results obtained with the agar diffusion method. (A) Antimicrobial activity of 3D chitin-Ag/AgBr scaffold (1), chitinous scaffold before metallization (2) against *E. coli*. Lack of antimicrobial activity has been observed by using of "Suprasorb® A + Ag" material (3). (B). The antimicrobial activity of 3D chitin-Ag/AgBr scaffold (1) against *B. subtilis*.

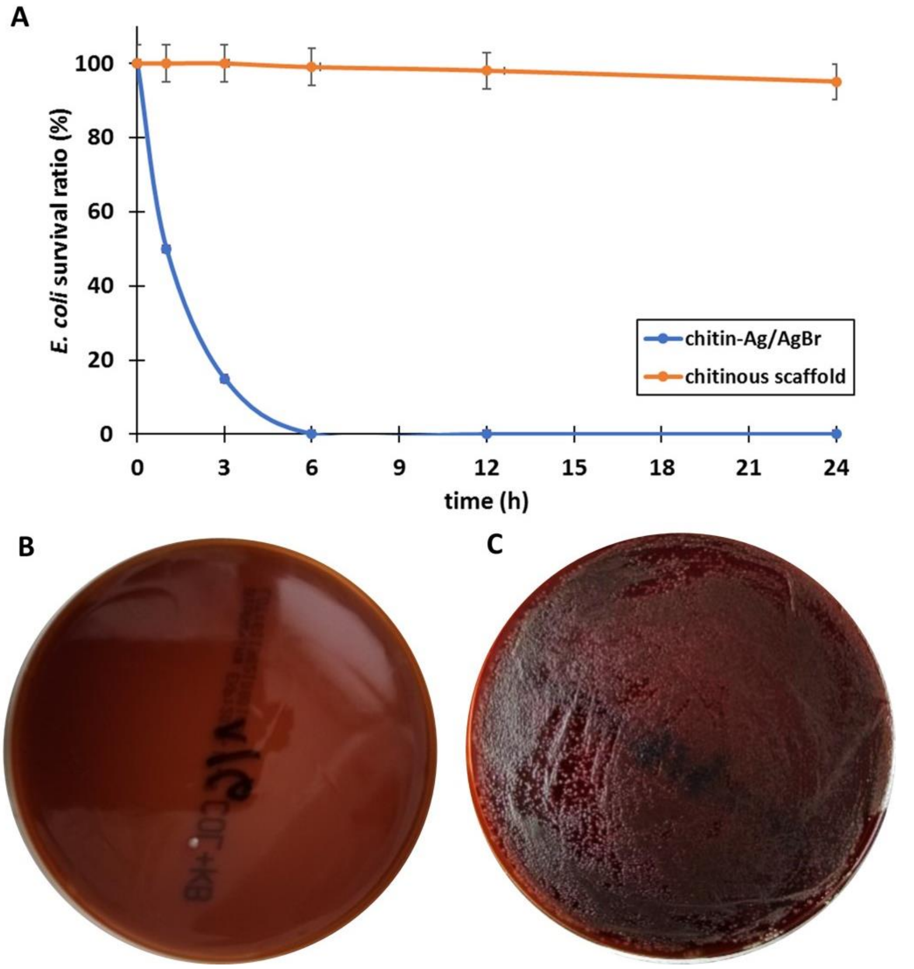

Figure S4. Dynamics of the degradation of live bacteria colonies as a function of time of filtration with 5% error bars (A). Filtration clearly indicate that 3D chitin-Ag/AgBr scaffold possess antibacterial properties against *E. coli* (ATCC® 25922) strain (blue line). Br-containing chitinous scaffold before AgNPs coating did not reflect any antibacterial effect. The number of survived bacteria's colony was uncountable, even after 24 h (orange line). Only one colony of *E. coli* survived after 24 h of filtration using chitin-Ag/AgBr scaffold (B). Br-containing chitinous scaffold without silver coating did not show antibacterial activity against *E. coli* even after 24 h (C).

Table S1. Results of 3D quantitative analysis (Micro-CT).

Material	Porosity	Average pore size (± SD)	Average fiber size (± SD)
chitinous scaffold (2)	98.5%	1350 μm (± 400 μm)	72 μm (± 17 μm)
chitin-Ag/AgBr scaffold (3)	98.2%	1300 μm (± 300 μm)	71 μm (± 18 μm)

Table S2. Results of local chemical analyses (SEM-EDX) of selected areas of the *A. aerophoba* chitinous scaffold after metallization.

**Element**	**At %**
**C**	28.1
**O**	26.3
**Mg**	1.8
**Si**	2.3
**S**	0.5
**Cl**	0.8
**Ag**	32.9
**Br**	7.1
